# Determining the optimal harvest time for pomegranate variety wonderful in semi-arid climate

**DOI:** 10.1038/s41598-025-92015-7

**Published:** 2025-03-05

**Authors:** Vahideh Narjesi, Alireza Bonyanpour, Ali Akbar Ghasemi-Soloklui

**Affiliations:** 1 Crop and Horticultural Science Research Department, Markazi Agricultural and Natural Resources Research and Education Center (AREEO), Arak, Iran; 2 Crop and Horticulture Science Research Department, Fars Agricultural and Natural Resources Research and Education Center, Agricultural Research, Education and Extension Organization, Shiraz, Iran; 3 Nuclear Agriculture Research School, Nuclear Science and Technology Research Institute (NSTRI), P.O. Box 31485498, Karaj, Iran

**Keywords:** Fruit mature, Fruit quality, Biochemical properties, Antioxidant activity, Field trials, Plant physiology, Plant reproduction, Ecology, Plant sciences

## Abstract

Due to limited local knowledge regarding the optimal harvest time for this non-native variety, a two-year study (2021–2022) was conducted using a randomized complete block design with four blocks. This study aimed to determine the ideal harvest time based on quantitative and qualitative fruit characteristics in saveh, which has a semi-arid climate. Twelve similarly sized trees were selected for each orchard, and fruits were harvested at three-time intervals: 155 days after flowering (DAF) (September 27), 170 DAF (October 12), and 185 DAF (October 27). Ten fruits from four sides of the tree canopy were collected and analyzed for physical and biochemical properties. The results showed that harvest time significantly affected fruit weight, aril weight, and juice percentage positively, while it negatively impacted rind percentage. The first harvest date yielded the lowest quantitative and qualitative traits, with incomplete skin and aril coloration. By the third harvest, pomegranate fruits exhibited the highest total soluble solids (17.76 °Brix), pH (3.41), and anthocyanin content (32.56 mg/L), along with the lowest total phenols (17.28 mg GAE/L), antioxidant capacity (79.78%), and titratable acidity (1.11%), resulting in the highest flavor or ripening index (16.31). In addition, cracking rates increased substantially, reaching 30.25% by the third harvest, compared to negligible levels of 20.72% by the second harvest. Juice percentage and aril weight improved significantly with delayed harvest, peaking on October 27. These findings suggest that October 12–27 is the optimal harvest window for superior fruit quality while considering the risk of fruit cracking. This study provides practical insights into harvest timing for maximizing the marketability and nutritional value of ‘Wonderful’ pomegranates in semi-arid climates.

## Introduction

Iran is one of the world’s leading producers of pomegranates, and it is recognized for its diverse and high-quality cultivars^1^. Native varieties, such as Mallas Momtaz Saveh, Alak Saveh, and Yousef Khani, have significantly contributed to the country’s reputation. Recently, the Wonderful variety, a globally recognized commercial cultivar, has been introduced to several pomegranate-growing regions of Iran, including the Saveh and Fars provinces^2^. The adoption of this variety diversifies and enhance the pomegranate industry in these regions. However, comprehensive studies on the performance of the Wonderful variety under Iran’s semi-arid climate, particularly regarding optimal harvest timing, are currently limited.

The pomegranate (*Punica granatum* L.**)**is a fruit crop native to Iran and the Himalayas. It thrives in semi-arid and Mediterranean climates. It is cultivated in various parts of the world, including Spain, Egypt, Russia, France, China, Japan, and the United States^3^. Among the many commercial varieties, the Wonderful variety, which was originally bred in Florida, is widely popular in the U.S. and Europe. This cultivar is known for its large fruit size, thick red rind, juicy arils, and balanced sweet-tart flavor, making it highly desirable for fresh consumption and export^4^.

The Wonderful variety exhibits specific climatic requirements for optimal growth and fruit quality. It thrives under hot days and cool nights, with a growing period from flowering to harvest of 160 to 190 days^5^. Wonderful pomegranates yield from the third year and commercial quality by the fifth or sixth year^6^. Cool weather during ripening, combined with significant diurnal temperature variations, enhances skin and aril coloration. While the variety is adaptable to a range of climatic, soil, and water conditions, its performance is significantly influenced by regional factors, such as temperature, rainfall, and soil type^5^. Since 2018, studies on the adaptability and harvest timing of the Wonderful variety have been conducted in various parts of Iran to optimize its yield and quality.

Harvest timing plays a pivotal role in determining the quality, storage potential, and marketability of pomegranate fruits^7^. Harvest timing is crucial for fresh and stored consumption. Studies have shown that cultivar, region, and harvest timing affect pomegranate quality indices. Physiological, biochemical, and structural changes during ripening enhance marketability in terms of taste, color, and size^8^. Physiological and biochemical changes during ripening influence key quality attributes, including size, skin, and aril color, total soluble solids (TSS), titratable acidity (TA), and the sugar-to-acid ratio^9^. Premature harvests often result in fruit with suboptimal color, flavor, and market potential, whereas delayed harvests can lead to overripe fruits, reduced clarity of arils, and compromised storage quality^10,11^. Premature harvests often result in fruit with suboptimal color, flavor, and market potential, whereas delayed harvests can lead to overripe fruits, reduced clarity of arils, and compromised storage quality^12,13^. Key ripeness indicators, such as TSS (15–17%), TA (< 2%), and the sugar-to-acid ratio, are commonly used for Wonderful pomegranates^14,15^.

Harvesting at the appropriate stage is crucial for achieving the highest levels of beneficial compounds, including anthocyanins, phenolic content, antioxidant activity, and resistance to cold damage^16,17^. Several studies have highlighted the impact of harvest timing on pomegranate quality. For example, GÖZLEKÇİ, et al.^18^ reported that fruit diameter increases during growth, whereas Nuncio-Jáuregui et al.^19^reported significant changes in taste, color, and soluble solids during ripening. Other studies reported that higher antioxidant activity corresponds with increased total anthocyanin and phenolic content during ripening^20^. Attanayake et al.^21^observed significant effects of maturity on external and internal fruit characteristics, whereas Kilic and Koyuncu^22^ found that phenolic and volatile compounds decrease with ripening, whereas antioxidants increase. Moreover, Jayarathne et al.^23^ reported that later season harvest increased the soluble solid content of the three varieties.

Given the growing significance of the Wonderful variety in Iran, this study aimed to evaluate its physical and biochemical properties across three harvest dates in two regions of Saveh. The primary objective of this study was to determine the optimal harvest time to achieve maximum fruit quality and marketability under semi-arid conditions. By aligning harvest timing with the variety’s ripening indicators and regional climate characteristics, this research provides valuable insights for pomegranate growers in Iran and similar semi-arid regions.

## Results

### The results of analysis of variance (ANOVA)

The combined analysis of variance (ANOVA) of the physical traits of the Wonderful pomegranate variety across years and locations is presented in Table 1. The results revealed significant effects of year on juice percentage and aril color and significant effects of location on fruit weight, aril weight, rind color, and aril color. However, the interaction between year and location was not significant for any physical traits.

**Table 1:** Combined analysis of variance for physical traits of wonderful pomegranate fruit across two years and two locations.

Source of Variation	df	Fruit weight (g)	Aril weight (g)	Rind %	Juice %	Length to diameter ratio	Rind color	Aril color
Year (Y)	1	343.58^ns^	348.009^ns^	82.04^ns^	65.95*	0.002^ns^	0.288^ns^	1.254^**^
Location (L)	1	79332.92^**^	15032.8^**^	75.79^ns^	9.12ns	0.001^ns^	1.216*	6.613^**^
Y * L	1	291.18^ns^	4.89^ns^	0.001^ns^	18.92ns	0.001^ns^	0.211ns	0.172^ns^
Error type 1	12	5236.48	2550.85	55.57	10.61	0.002	0.139	0.493
Harvest time (HT)	2	17218.18^**^	13768.27^**^	135.71^**^	515.39**	0.023**	43.62**	16.11**
HT * Y	2	140.204^ns^	10.55^ns^	0.423^ns^	10.34^ns^	0.001^ns^	0.315^ns^	0.118^ns^
HT * L	2	342.089^ns^	402.67^ns^	16.57^ns^	118.64**	0.005*	3.82**	0.187^ns^
HT * Y * L	2	100.37^ns^	0.978^ns^	3.324^ns^	8.74^ns^	0.001^ns^	0.148ns	0.111^ns^
Error type 2	24	1959.45	1842.39	24.29	10.29	0.002	0.174	0.194
CV%		14.2	16.7	12.9	13.61	4.7	19.45	22.03

^ns^, ^*^ and ^**^ indicate non-significant, and significant at 5% and 1% probability levels, respectively.

Harvest time had a highly significant effect (*p* ≤ 0.01) on all physical traits of the Wonderful pomegranate fruit. Additionally, the interaction between harvest time and location was significant for juice percentage and rind color (*p* ≤ 0.01) and length-to-diameter ratio (*p* ≤ 0.05). However, the interaction effects of harvest time with year and the three-way interaction of harvest time, year, and location were not significant for any traits. Based on the non-significant interactions involving the year, mean comparisons for physical traits were performed separately for each location using averaged data over the two years, as shown in Table 1.

Orchard A: In Orchard A, harvest time significantly influenced all physical traits (Table 1). Fruits harvested on October 27 exhibited the highest values for fruit weight, aril weight, and juice percentage, along with the most intense rind and aril colors (Table 2). This suggests that a later harvest date allows for better fruit development and ripening. Conversely, fruits harvested on September 27 exhibited the lowest values for these traits, indicating incomplete ripeness at this stage.

**Table 2:** Effect of harvest time on physical traits of wonderful pomegranate fruit in orchard A (Average data over 2 years).

Harvest time	Fruit weight (g)	Aril weight (g)	Rind (%)	Juice (%)	Length to diameter ratio	Rind color	Aril color
First harvest (155 DAF)	289.02b	166.83b	43.75a	29.84c	0.919a	3.49c	2.73c
Second harvest (170 DAF)	310.44a	197.33a	41.83ab	35.97b	0.902ab	5.07b	3.31b
Third Harvest (185 DAF)	327.17a	203.84a	38.69b	39.26a	0.895b	5.48a	3.85a

**ns, * and ** indicate non-significant, significant at 5% and 1% probability levels, respectively.

Orchard B: In Orchard B, similar trends were observed (Table 3). Harvest time significantly affected all physical traits, except rind percentage. Fruits harvested on October 27 had the highest fruit and aril weight, juice percentage, and most intense rind and aril color. As observed for Orchard A, delaying harvest until late October resulted in superior fruit quality attributes.

**Table 3:** Effect of harvest time on physical traits of wonderful pomegranate fruit in orchard B (average data over 2 years).

Harvest time		Fruit weight (g)	Aril weight (g)	Rind (%)	Juice (%)	Length to diameter ratio	Rind color	Aril color
First harvest (155 DAF)		339.4b	193.94b	44.03a	33.89b	0.941a	4.08c	2.35c
Second harvest (170 DAF)		351.23b	214.16a	41.39a	35.16b	0.917a	4.61b	2.73b
Third Harvest (185 DAF)		376.83a	221.19a	42.2a	37.49a	0.877b	5.91a	3.52a

ns, * and ** indicate non-significant, significant at 5% and 1% probability levels, respectively.

These findings highlight the critical role of harvest timing in determining the physical traits of pomegranate fruits. The third harvest date (October 27) consistently resulted in the best outcomes for both orchards, with significant improvements in fruit weight, juice content, and color intensity. These results suggest that late October is the optimal harvest period for Wonderful pomegranates in semi-arid climates, enabling growers to achieve maximum fruit quality and marketability.

### Fruit weight

Significant differences in fruit weight were observed at different harvest times in both regions. As the harvest was delayed, fruit weight showed an increasing trend, with this increase varying between Orchards A and Orchard B (Tables 2 and 3). The highest fruit weight was recorded at the third harvest of Orchard A (327.17 g). The second harvest had the next highest fruit weight (310.44 g), with no significant difference from the third harvest. The lowest fruit weight was observed in the first harvest (289.02 g) (Table 2). In Orchard B, the highest fruit weight was recorded in the third harvest (376.83 g) and the lowest weight was recorded in the first harvest (339.4 g) (Table 3). These results suggest that delaying harvest allows for greater fruit development and biomass accumulation, leading to heavier fruits.

### Aril weight

In both orchards, significant differences were observed in aril weight across different harvest times. Delaying the harvest increased the aril weight. In Orchard A, the aril weight significantly increased from the first to the second and third harvests. The highest aril weight in Orchard A was recorded in the third harvest (203.84 g), followed by the second harvest (197.33 g) and the first harvest (166.83 g) (Table 2). A similar increasing trend was observed in Orchard B, where the aril weight increased from the first to third harvests. However, the increase between the second and third harvests was not significant. The highest aril weight was recorded in the third harvest (221.19 g) and the lowest in the first harvest (193.94 g) (Table 3). This trend indicates that aril development continues as the fruit matures, which contributes to the overall quality of the fruit.

### Rind percentage

The percentage of rinds decreased as the fruit matured. In Orchard A, significant differences in rind percentage were observed between different harvest times at the 5% significance level. The highest rind percentage in orchard A was recorded at the first harvest (43.75%), and the lowest at the third harvest (38.69%) (Table 2). In Orchard B, no significant differences in rind percentage were observed across different harvest times, with the highest and lowest rind percentages recorded in the first (44.03%) and second (41.39%), respectively (Table 3). These findings suggest that as the fruit matures, the relative amount of rind decreases, which is beneficial for juice production and consumer preference.

### Juice percentage

Significant increases in the percentage of juice were observed with delayed harvest times in both regions. In Orchard A, the highest percentage of juice was recorded in the third harvest (39.26%), whereas the lowest percentage was recorded in the first harvest (29.84%) (Table 2). In Orchard B, significant differences in the juice percentage were observed between the first and third harvests. The highest percentage of juice was produced in the third harvest (37.49%), and the lowest percentage was produced in the first harvest (33.89%) (Table 3). These results indicate that delaying the harvest increases the juice content of the fruits, enhancing their commercial value for fresh consumption and juice production.

### Length to diameter ratio

Harvest time had a significant effect on the length-to-diameter ratio in Orchard A. However, the ratio decreased as the fruit matured. The highest length-to-diameter ratio was observed in the first harvest (0.919) and the lowest in the third harvest (0.895) (Table 2). In Orchard B, a significant decrease in the length to diameter ratio was observed during fruit maturation. The highest and lowest ratios were recorded in the first (0.941) and third (0.877) harvests, respectively (Table 3). These trends showed that the fruit becomes more rounded as it matures, which may indicate optimal ripeness.

### Rind color

**Fig. 1:**
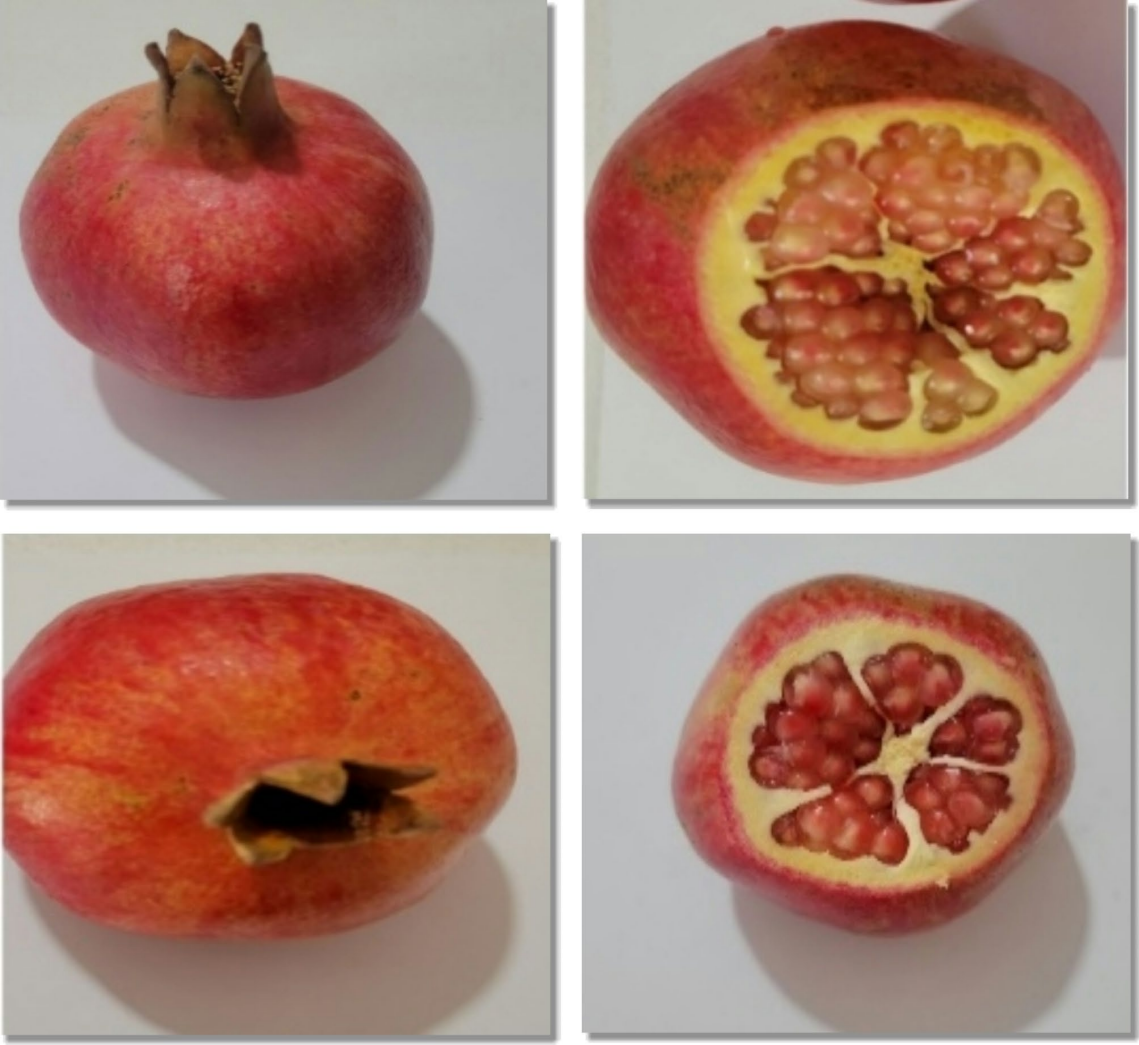
Rind and aril color of Wonderful pomegranate at the first harvest (Up: Orchard A, Down: Orchard B).

**Fig. 2:**
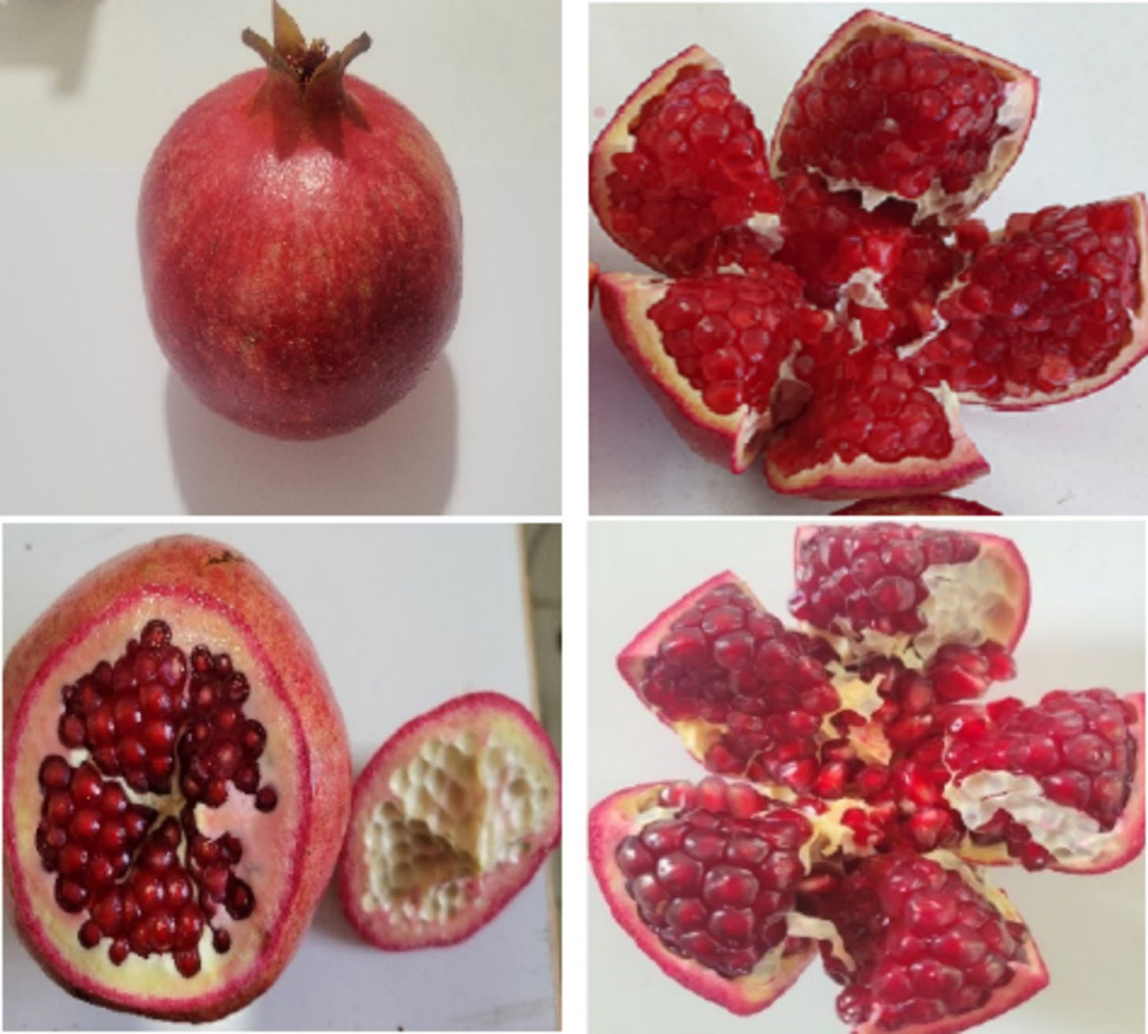
Rind and aril color of Wonderful pomegranate at the second harvest (Up: Orchard A, Down: Orchard B).

**Fig. 3:**
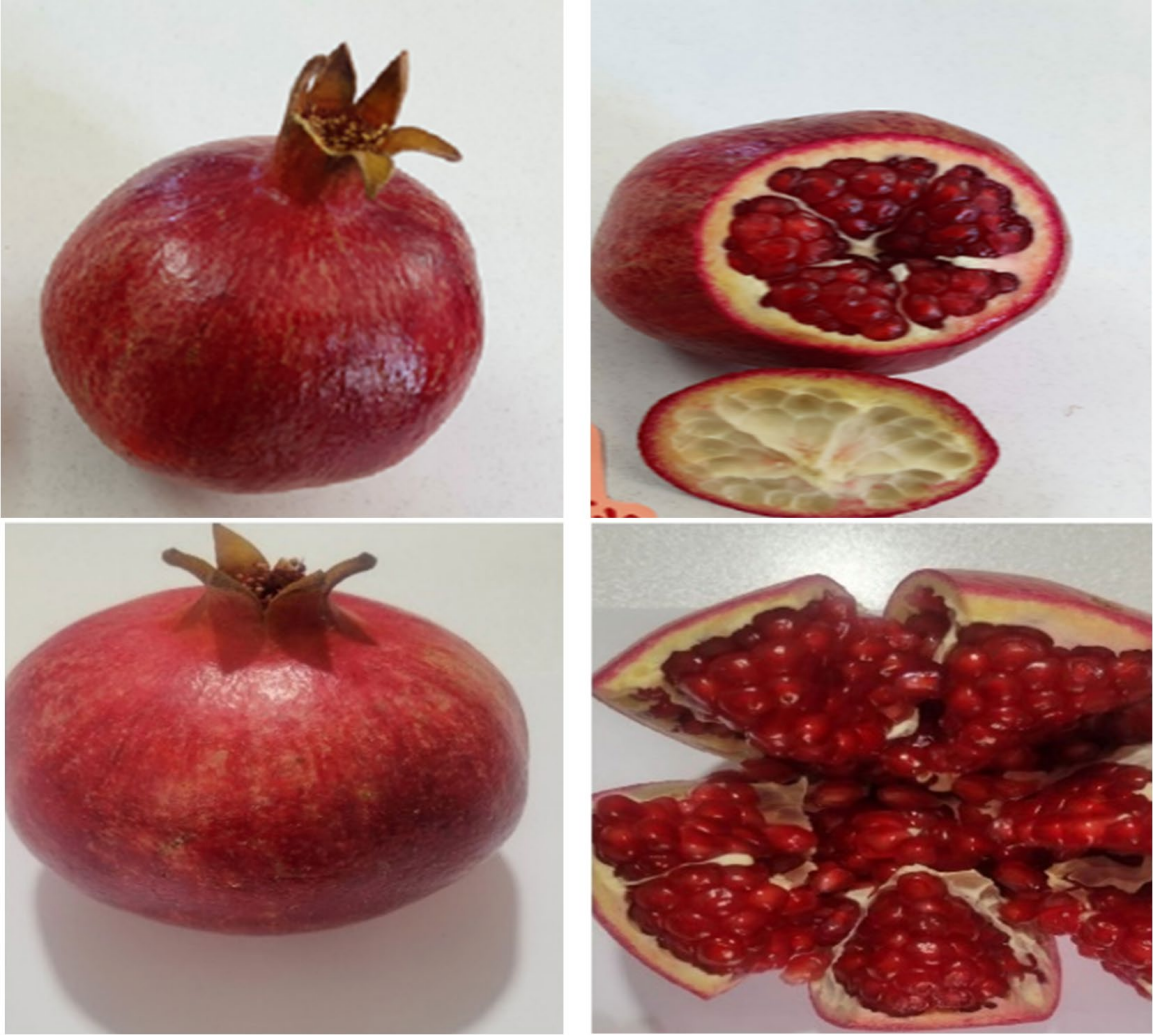
Rind and aril color of Wonderful pomegranate at the third harvest (Up: Orchard A, Down: Orchard B).

 Significant differences in rind color were observed between harvest times in both regions (Figs. 1, 2 and 3). The rind color of the fruit intensifies as the harvest is delayed. In Orchard A, the highest rind color value (Fig. 3) was recorded in the third harvest (5.48), and the lowest index of color (Fig. 1) was recorded in the first harvest (3.49) (Table 2). Similarly, in Orchard B, rind color increased significantly with delayed harvests, with the highest value recorded in the third harvest (5.91) and the lowest value recorded in the first harvest (4.08) (Table 3). This indicates that mature fruits develop a more attractive and intense rind color, which is desirable for marketability.

**Fig. 4:**
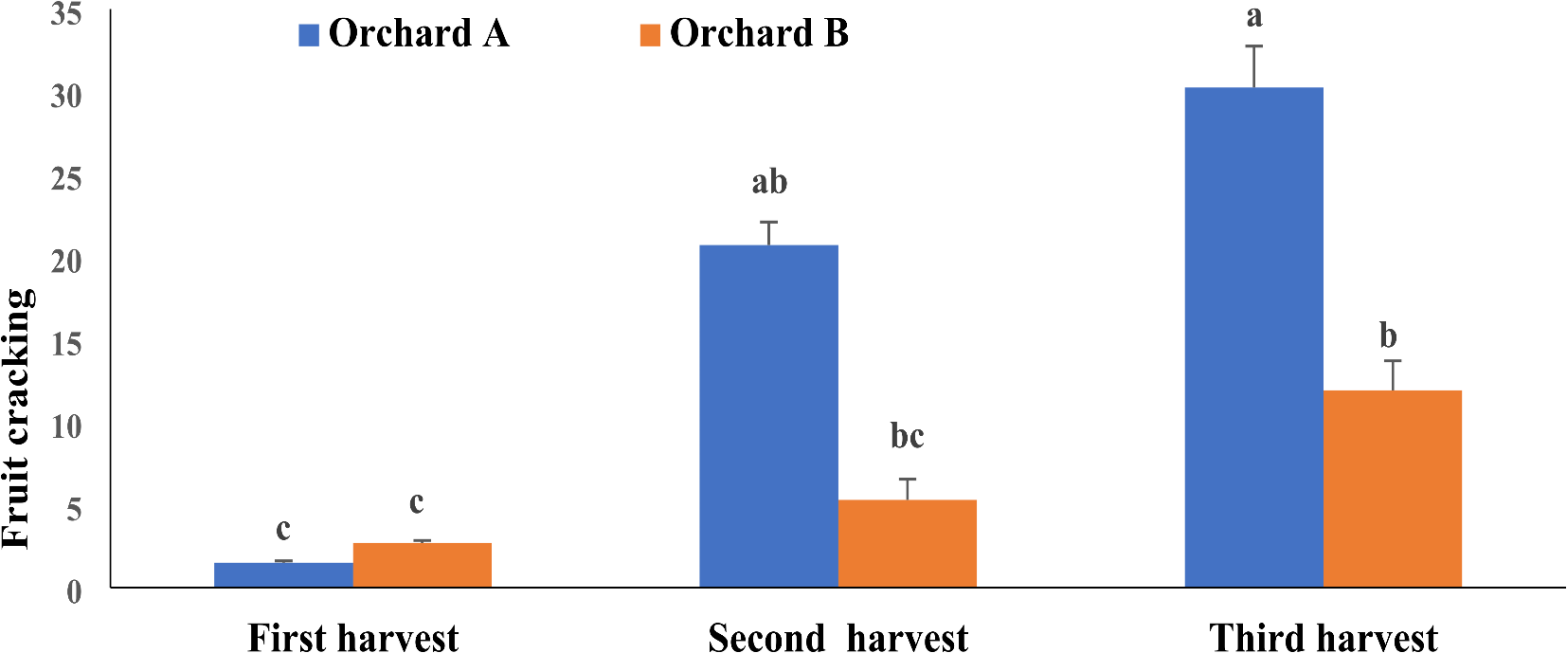
Effect of harvest time on fruit cracking percentage of Wonderful pomegranate fruit in different location (Average data over 2 years).

### Aril color

Significant differences in aril color were observed at different harvest times. The aril color of the fruit matured, aril color increased (Figs. 1, 2 and 3). In Orchard A, significant differences in aril color were observed between harvest times. The highest aril color value was recorded in the third harvest (3.85) and the lowest in the first harvest (2.73) (Table 2). In Orchard B, aril color increased with delayed harvest times, with the highest value recorded in the third harvest (3.52) and the lowest value recorded in the first harvest (2.35) (Table 3). This suggests that the arils become more visually appealing and likely to become more flavorful as they mature.

The harvest timing significantly affects the physical traits of pomegranate fruits. Delaying harvest until October 27 resulted in the highest fruit weight, aril weight, juice percentage, and most intense skin and aril color in both orchards. These findings showed that delaying harvest until late October can significantly enhance the quality and marketability of pomegranate.

### Effect of harvest time on the quality and biochemical traits of pomegranate juice

Based on the combined analysis of variance (ANOVA) of the data (Table 4), the main effect of the year was significant for all traits, except the flavor index. The main effect of location was significant for all biochemical traits. The interaction effect between year and location was not significant for any traits except pH. Harvest time significantly affected the quality and biochemical traits of the pomegranate juice. The interaction effects of harvest time with year and the three-way interaction of harvest time, year, and location were not significant for any traits. However, the interaction between harvest time and location was significant for total soluble solids and total anthocyanin at the 1% level and for pH and titratable acidity at the 5% level (Table 4). Given the non-significant interactions of harvest time with year and the three-way interaction with year and location, mean comparisons for these traits were performed separately for each location using average data over the two years.

**Table 4:** Combined analysis of variance for quality and biochemical traits of wonderful pomegranate juice across 2 years and locations.

SOV	df	pH	Total soluble solids (Brix)	Titratable acidity	Flavor index	Total anthocyanin (mg/L)	Antioxidant capacity (%)	Total phenol (mg GAE/L)
Year (Y)	1	0.352**	2.126*	0.345*	6.172ns	108.316**	173.186**	118.447**
Location (L)	1	0.056**	28.71**	7.614**	751.574**	31.33**	29.9**	17.65**
Y * L	1	0.281**	0.797ns	0.21ns	4.64ns	0.371ns	15.45ns	1.412ns
Error type 1	12	0.015	1.38	0.152	7.024	5.65	2.311	2.299
Harvest time (HT)	2	0.216**	169.54**	1.036**	244.99*	2424.097**	551.86**	930.67**
HT * Y	2	0.012ns	0.116ns	0.023ns	1.93ns	1.345ns	0.851ns	10.88ns
HT * L	2	0.027*	6.18**	0.228*	4.49ns	10.009**	1.78ns	0.05ns
HT * Y * L	2	0.031ns	0.057ns	0.027ns	1.61ns	3.822ns	1.44ns	0.996ns
Error type 2	24	0.013	0.528	0.037	2.09	2.505	1.54	1.62
CV%		3.87	11.27	25.25	2.96	24.19	3.97	14.62

ns, * and ** indicate non-significant, significant at 5% and 1% probability levels, respectively.

### Total soluble solids (TSS)

Significant differences in TSS were observed among the three harvest times in both regions, with a significant increase in TSS as the harvest was delayed (Tables 5 and 6). In Orchard A, the highest TSS (17.76 Brix) was recorded in the juice from fruits harvested on October 27, followed by the juice from fruits harvested on October 20 (15.23 Brix), and the lowest TSS (13.34 Brix) was observed in the juice from the first harvest on October 5 (Table 5). In Orchard B, the highest TSS was observed in the juice from the third harvest (18.02 Brix), and the lowest (15.00 Brix) was observed in the juice from the first harvest (Table 6). These findings indicate that delaying the harvest allows the fruits to accumulate more sugar, enhancing their sweetness and overall flavor.

**Table 5:** Effect of harvest time on quality and biochemical traits of wonderful pomegranate juice in orchard A (average data over 2 years).

Harvest time	Total phenol (mg GAE/L)	Antioxidant capacity (%)	Total anthocyanin (mg/L)	pH	Flavor index	Titratable acidity (%)	Total soluble solids (°Brix)
First harvest (155 DAF)	25.14a	85.27a	17.07c	3.25b	6.86c	1.921a	13.34c
Second harvest (170 DAF)	18.98b	80.92b	26.35b	3.26b	9.41b	1.611b	15.23b
Third Harvest (185 DAF)	16.64c	78.55c	30.79a	3.41a	11.26a	1.529b	17.76a

ns, * and ** indicate non-significant, significant at 5% and 1% probability levels, respectively.

**Table 6:** Effect of harvest time on quality and biochemical traits of wonderful pomegranate juice in orchard B (average data over 2 years).

Harvest time	Total phenol (mg GAE/L)	Antioxidant capacity (%)	Total anthocyanin (mg/L)	pH	Flavor index	Titratable acidity (%)	Total soluble solids (°Brix)
First harvest (155 DAF)	25.87a	86.28a	18.13c	3.30b	11.66c	1.306b	15.00c
Second harvest (170 DAF)	19.73b	81.41b	26.30b	3.34b	13.29b	1.26a	15.97b
Third Harvest (185 DAF)	17.28c	79.78c	32.56a	3.40a	16.31a	1.115b	18.02a

**ns, * and ** indicate non-significant, significant at 5% and 1% probability levels, respectively.

### Titratable acidity (TA)

Significant differences in TA were observed among the three harvest times in both regions at the 1% significance level (Tables 5 and 6). TA decreased with the delay in harvest time. The highest TA in Orchard A (1.921%) and Orchard B (1.306%) was recorded in the juice from the first harvest on October 5. The lowest TA values in Orchard A (1.529%) and Orchard B (1.115%) were recorded in the juice from the third harvest of October 27. The decrease in acidity with delayed harvesting contributes to a sweeter taste and improved flavor balance in the juice.

### Flavor index (TSS/TA)

The flavor index, which is the ratio of TSS to TA, increased with the delayed harvest time. The highest flavor index was observed in the juice from the third harvest in both regions (Tables 5 and 6). In Orchard A, the lowest flavor index was recorded in the juice from the first harvest (6.86), whereas the highest flavor index was recorded in the juice from the third harvest (11.26) (Table 5). In Orchard B, the lowest flavor index was recorded in the juice from the first harvest (11.66), and the highest flavor index was recorded in the juice from the third harvest (16.31) (Table 6). This indicates that fruits harvested later have a better sugar-to-acid ratio, making them more palatable.

### pH

Significant differences in pH were observed among the three harvest times in both regions. The pH increased with delayed harvest time. The lowest pH was recorded in the juice from the first harvest in both regions (3.25 in Orchard A and 3.30 in Orchard B), whereas the highest pH was recorded in the juice from the third harvest (3.41 in Orchard A and 3.40 in Orchard B) (Tables 5 and 6). The increase in pH after delayed harvesting is associated with a reduction in acidity and improved taste.

### Total anthocyanin

Significant differences anthocyanin content was observed among harvest times in both regions at the 1% significance level (Tables 5 and 6). Total anthocyanin content increased with delayed harvest time. The lowest total anthocyanin content was recorded in the juice from the first harvest (17.07 mg/L in Orchard A and 18.13 mg/L in Orchard B), and the highest was recorded in the juice from the third harvest (30.79 mg/L in Orchard A and 32.56 mg/L in Orchard B). Higher anthocyanin content indicates better color development and potential health benefits due to the antioxidant properties of anthocyanins.

### Antioxidant capacity

Antioxidant capacity, measured as the percentage of DPPH radical scavenging activity, decreased with delayed harvest time. The highest antioxidant capacity was recorded in the juice from the first harvest in both regions (85.27% in Orchard A and 86.28% in Orchard B), whereas the lowest was recorded in the juice from the third harvest (78.55% in Orchard A and 79.78% in Orchard B) (Tables 5 and 6). The decrease in antioxidant capacity with delayed harvest suggests a trade-off between increased sugar content and reduced antioxidant properties.

### Total phenolic compounds

Significant differences phenol content was observed between harvest times in both regions. Total phenol content decreased with delayed harvest time. The highest total phenol content was recorded in the juice from the first harvest in both regions (25.14 mg GAE/L in Orchard A and 25.87 mg GAE/L in Orchard B), and the lowest was recorded in the juice from the third harvest (16.64 mg GAE/L in Orchard A and 17.28 mg GAE/L in Orchard B) (Tables 5 and 6). Phenolic compounds contribute to the antioxidant capacity and potential health benefits of pomegranate juice.

### Fruit cracking percentage

The results of this study indicate significant differences in fruit cracking percentage at different harvest times (Fig. 4). The percentage of fruit cracking increased as the harvest period was prolonged and the growth and development cycle. In both regions, the percentage of fruit cracking was negligible at the first harvest on October 5. In Orchard A, 20.72% of the fruits were cracked by the second harvest on October 12 and 30.25% by the third harvest on October 27. In Orchard B, 5.3% of the fruits were cracked by the second harvest on October 12 and 11.93% by the third harvest on October 27.

The harvest timing significantly affects the quality and biochemical traits of pomegranate juice. Delaying the harvest until October 27 resulted in the highest pH, total soluble solids, flavor index, total anthocyanin, antioxidant capacity, and total phenol content, and the lowest titratable acidity in either orchard. These findings suggest that delaying harvest until late October can significantly enhance the nutritional and commercial value of Wonderful pomegranates. However, the increased risk of fruit cracking associated with delayed harvest should be considered. Optimal harvest timing should balance quality improvements with the risk of fruit cracking.

## Discussion

This study evaluated the effects of harvest timing on the physical and biochemical traits of the Wonderful pomegranate variety grown in Saveh, which has a semi-arid climate. Significant variations in traits across years and locations highlight the role of microclimatic factors, including temperature fluctuations and rainfall patterns. These findings align with those of previous studies, such as those by Tarantino, et al.^24^and Caliskan and Bayazit^25^ which emphasize the impact of cultivar and ecological conditions on the physical and biochemical traits of pomegranate varieties. Mphahlele et al.^26^ also noted that the Wonderful cultivar exhibits different characteristics when grown in different geographical areas.

### The effect of harvest time on physical traits

Delaying harvest time significantly increased fruit weight, aril weight, and juice percentage. This trend is in agreement with previous reports, such as Rashno Nuzhad, et al.^27^ and Meighani, et al.^28^ who observed similar increases in fruit and aril weight in late-harvested fruits. Moreover, GÖZLEKÇİ, et al.^18^reported that the fruit weight of the “Hicaznar” pomegranate variety in Antalya increases as the fruit matures^29^. noted that larger fruits have significantly heavier arils, related to increased fruit size during maturation. In addition, the rind percentage decreased with delayed harvests as the aril weight became more dominant, consistent with observations by Amiryousefi, et al.^30^. The length-to-diameter ratio, an important indicator of fruit shape, decreased with delayed harvests, indicating a shift in fruit development dynamics. This was corroborated by Boussaa et al.^31^, who found significant changes in fruit dimensions across harvest times. Rind and aril coloration, which are critical quality markers, improved significantly with delayed harvests. Enhanced redness in arils, noted as desirable for both fresh consumption and processing^38^, indicates advanced maturity and is consistent with findings for other pomegranate varieties^32^.

### The effect of harvest time on the quality and biochemical trait

Key quality indicators, including TSS, TA, and flavor index, showed significant changes across harvest times. As the harvest was delayed, TSS levels increased, whereas TA and acidity decreased. This aligns with findings by Boussaa, et al.^31^ and Rashno Nuzhad, et al.^27^ who reported that delayed harvesting enhances sugar content due to starch hydrolysis during maturation. Kashash et al.^4^ noted that starch hydrolysis into simple sugars during fruit maturation increases the sugar content, ranging from 12 to 16% in fully mature pomegranates. Differences in TSS could be due to variations in harvest timing, growing season, geographical conditions, and cultivar types. Fernandes et al.^33^observed significant differences in TSS content among nine pomegranate cultivars, with values ranging from 14.8°Brix to 18.04 °Brix. Moreover, another study showed that early-harvested fruits have higher acidity, making them less suitable for fresh consumption^31^. Tehrani et al. (2011) reported that reduced total acidity results from the use of organic acids in metabolic activities. Fawole^32^ found that TA levels decrease during fruit maturation because organic acids are used as respiratory substrates and carbon skeletons for new compound synthesis.

The pH of Wonderful pomegranate juice increased with delayed harvests, consistent with the findings of Fawole and Opara^34^, who reported increased pH and decreased acidity in three pomegranate varieties in South Africa during maturation. The pH indicates the concentration of H + ions, which affect the acidic taste of the juice^35^. The total anthocyanin content was highest in fruits from the latest harvest, consistent with the findings of Borochov-Neori et al.^36^ who noted that anthocyanin levels peak as fruits approach full maturity. Anthocyanin synthesis is closely linked to the degradation of phenolic compounds, as suggested by Tabar et al.^37^. Anthocyanin stability varies with temperature, pH, light, and oxygen, and it is sensitive to oxidative enzymes^38^. This explains the observed decrease in total phenolic content with delayed harvests, a trend also reported by Rashno Nuzhad et al.^27^. While phenolic content contributes to antioxidant capacity, its reduction during maturation suggests a trade-off between antioxidant properties and sweetness in late-harvested fruits^32^.

### The effect of harvest time on fruit cracking

Fruit cracking was significantly influenced by harvest time, particularly in Orchard A, where delayed harvesting increased the incidence of cracking. This phenomenon can be attributed to a combination of reduced rind thickness and increased aril weight during maturation, which increases the susceptibility to cracking under fluctuating temperatures. Similar findings were reported by Abd El et al.^39^ who highlighted the risks associated with delayed harvests in terms of fruit cracking and storage quality.

### Balancing quality and harvest timing

While delayed harvesting improves key physical and biochemical traits, such as fruit weight, aril weight, juice percentage, TSS, pH, and anthocyanin content, it also increases the risk of fruit cracking. These trade-offs underscore the importance of optimizing harvest timing to balance improved fruit quality with reduced postharvest losses. For pomegranates in Saveh, harvesting around October 27 offers the highest quality and marketability, although growers should remain vigilant about the increased risk of cracking and its implications for storage and transport.

## Conclusion

With delayed harvests producing better fruit quality, the results showed notable impacts of harvest time on fruit weight, aril weight, juice percentage, and color intensity. Specifically, despite showing the lowest titratable acidity, fruits picked on October 27 had the best values for total soluble solids, pH, and anthocyanin content. These biochemical enhancements highlight the possibility of maximizing the commercial and nutritious value of wonderful pomegranates with a careful harvesting schedule. The study also found a higher risk of fruit shattering associated with delayed harvests. Given both quality improvements and possible physiological problems, this emphasizes the requirement of a balanced approach to determine the ideal harvest time. The results coincide with earlier studies and provide a fresh understanding of the complex relationship between pomegranate fruit growth and harvest timing. For pomegranate growers and the larger agricultural community, this study has major pragmatic implications. Growers can maximize fruit quality and marketability by choosing the ideal harvest season—late October to early November—thereby reducing the risk of fruit cracking. Future studies should investigate the storage and post-harvest properties of wonderful pomegranates collected at various times to create recommendations for best practices in pomegranate farming and harvesting.

Finally, underlining the important role of harvest timing in improving fruit quality, this study makes significant contributions to pomegranate research. The results provide a basis for creating optimal harvest plans, thus guaranteeing that wonderful pomegranates will realize their full potential in local and worldwide markets.

## Materials and methods

### Experimental location

The study was conducted in two adapted orchards of the Wonderful pomegranate variety:

1. The Pomegranate Research and Education Campus in Saveh, Switzerland.
2. An orchard in the village of Estuj, located 30 km from the first orchard, was established in the Qara-Chai rural district of Saveh County.

The average elevation of Saveh city is 960 m above sea level, and the climate is semi-arid, with hot summers and slightly cold winters. Moreover, the meteorological information (monthly average) for 2021 and 2022 from the Saveh synoptic meteorological station present in Table 7. The geographical location, soil, and water characteristics of the experimental sites are summarized in Table 8.

**Table 7:** Meteorological information (monthly average) of the years 2021 and 2022 from the Saveh synoptic meteorological station.

	Average minimum temperature (C^º^)		Average maximum temperature (C^º^)		Average temperature (C^º^)		Average humidity (%)		wind speed (km/h)		Average hours of sunshine		Total monthly rainfall (mm)	
	2021	2022	2021	2022	2021	2022	2021	2022	2021	2022	2021	2022	2021	2022
March	11.3	11.4	24.9	23.3	18.1	17.3	28	33	28	-	8.2	8.1	1.1	12.9
April	15.7	15.8	28.8	29.7	22.3	22.8	28	24	27	-	8.8	10	8.4	0.8
May	21.7	22	35.6	35.5	28.6	28.8	16	25	20	25	11.6	10.1	0	5.5
June	25.2	25.3	40	39.7	32.1	32	16	21	18	16	12	11.7	0	0
July	25.7	26.3	39.3	39.2	32	32.5	21	18	18	16	11.6	11	5.9	0
August	21.4	22.9	35.2	35.7	28.3	29.3	15	22	18	16	10.9	10.7	0	0
September	17.9	15.6	30.9	27.5	24.4	21.6	19	31	15	19	9.4	8.6	0	1.9
October	8.9	11.5	20.8	23.2	14.9	17.3	38	38	18	21	7.4	7.1	8.2	1.1
November	4.4	4.8	13.9	17.4	9.1	11.1	42	36	14	18	4.7	7	0.5	0.3
December	−0.8	4	8.3	15.4	3.7	9.7	45	41	15	23	4.9	6.6	15.8	7.7
January	−0.3	3.7	9.1	13.1	4.4	8.4	46	51	22	25	6.1	6	57.9	46.1
February	8.5	4	19.7	13.4	14.1	8.7	40	51	21	16	7.6	5.5	9.8	10.8

**Table 8:** Geographical location, soil, and water characteristics of the experimental sites.

Orchard	Geographical location	Soil characteristics	Water characteristics
A	Central District of Saveh County, Noorali-Beyk rural district Latitude: 35° 0.664’ N; Longitude: 50° 21’ E	Loam soil; EC: 5.41 dS/m; pH: 7.1	Water source: well EC: 3.77 dS/m pH: 7.1
B	Central District of Saveh County, Qara-Chai rural district Latitude: 34° 53’ N; Longitude: 50° 22’ E	Loam soil; EC: 3.9 dS/m; pH: 7.7	Water source: well EC: 2.53 dS/m pH: 7.45

The locations of the two experimental orchards were geographically distinct, but both fell within the semi-arid climate zone typical of Saveh County, which supports pomegranate cultivation due to its hot summers and moderately cold winters. The soil in both locations was loamy, providing good drainage and fertility, which are essential for the healthy growth of pomegranate trees. The water quality for irrigation sourced from wells varies slightly between the two orchards in terms of electrical conductivity (EC) and pH, which can influence overall growth and fruit quality. These detailed geographical and environmental profiles provide the necessary context for interpreting the results of the adaptation and harvest timing studies on the Wonderful pomegranate variety in these specific regions.

### Experimental design

Two ‘Wonderful’ pomegranate orchards, each comprising five-year-old trees, located in different regions of Saveh, were selected for this study. To determine the optimal harvest time, a randomized complete block design with four replications was used over two years (2021–2022). Twelve similarly sized trees were selected from each orchard (three trees per replication). Fruits were harvested at three intervals: the first harvest at 155 days after flowering (DAF) (September 27), the second harvest at 170 DAF (October 12), and the third harvest at 185 DAF (October 27). Ten fruits were randomly picked from four sides of each tree canopy and analyzed for quantitative and qualitative traits.

### Measurement of quantitative and qualitative fruit traits

Fruits were collected and stored at 4 °C until analysis. Fruit length and diameter (mm) and rind thickness (mm) were measured using a vernier caliper.

Following measurements of the total fruit size, the fresh weight of the rind which includes both the outer red layer and the inner creamy layer, was determined using a digital scale with an accuracy of 0.001 g. The husks were carefully cut with a sharp knife in the equatorial zone after the fresh weight of the fruit was determined. To calculate the weight of the arils and rind percentage, the rind and arils were physically removed. Moreover, the juice percentage was calculated according to the method of Zarei et al.^40^. Fruit juice content was measured by extracting the total arils from each fruit using an electric extractor (Toshiba 5020) and the results are presented as percentages.

Fruit color was determined by extracting juice from a portion of the rind, which was then centrifuged at 5000 rpm for 10 min at 4 °C. The resulting juice was analyzed for color based on absorbance at 530 nm using a Helios Omega UV–vis spectrophotometer (Thermo Scientific Technologies, Madison, USA). The absorbance value correlated with the intensity of the rind color. Moreover, the aril color was assessed using a similar procedure. The juice from the arils was extracted and centrifuged at the same speed and temperature as the rind juice. The absorbance of the aril juice was measured at 530 nm to evaluate its color intensity and uniformity. This spectrophotometric method provided an objective and quantitative measure of color, which was used to classify the aril color based on its intensity. The methods used to estimate fruit and aril colors were adapted from Fawole and Opara^41^ methods.

Additionally, twelve trees in each region were harvested on the specified dates to determine the percentage of fruit cracking, which was calculated as the ratio of cracked fruits to total fruits (sum of healthy and cracked fruits).

### Measurement of the juice quality traits

Biochemical parameters such as pH, TSS, as well as TA, were assessed using fresh pomegranate arils. By extracting and combining one drop of juice from each fruit into a digital refractometer (Atago NI, Japan) at 20 °C, the total soluble solids (°Brix) in the juice were determined. The results were then displayed as a percentage. By titrating juice samples (5 mL) with 0.1 N NaOH to the titration endpoint of pH 8.2, which was tracked using a pH meter (Labtron), TA, which was expressed as citric acid concentration (g.100ml^−1^) was ascertained. At room temperature, the pH of the aril juice samples was measured and homogenized using a pH meter (WTW 526, Germany) calibrated to a pH range of 4–7^42^. The fruit flavor index was calculated as the ratio of TSS to TA (TSS/TA).

### Measurement of biochemical traits

The Folin-Ciocalteu (Folin-C) and colorimetric methods developed by Ghasemi-Soloklui, et al. 20 for pomegranate were used to assess the total phenol content in juice samples. Using a spectrophotometer set to 750 nm, the total phenol content in the juice sample was determined by applying the Folin-Ciocalteu reagent. The results were calculated using the mean value (mg) of gallic acid equivalents (GAEs) per milliliter of crude juice.

Total anthocyanin content was measured using the differential pH method, with absorbance readings at 520 and 700 nm for pH 1.0 and pH 4.5 buffers, respectively^43^. The anthocyanin concentration was calculated as follows:

$$\:\text{T}\text{o}\text{t}\text{a}\text{l}\:\text{A}\text{n}\text{t}\text{h}\text{o}\text{c}\text{y}\text{a}\text{n}\text{i}\text{n}=\left[\left(A\times\:MW\times\:DF\times\:100\right)/MA\right]$$

$$\:A=\left(A520-A700\right){pH}_{1}-\left(A520-A700\right){pH}_{4/5}$$

where MW (molecular weight of the predominant anthocyanin) = 440, DF (dilution factor) = 10, and MA (molar absorptivity of cyanidin-3-glucoside) = 26,900.

The antioxidant capacity of the juice was determined using the DPPH (2,2-diphenyl-1-picrylhydrazyl) radical scavenging method^44^. Absorbance was measured at 515 nm using a spectrophotometer, and antioxidant capacity was expressed as the percentage of DPPH inhibition: DPPHsc = b(a − b)​×100, where a is the absorbance of DPPH, and b is the absorbance of DPPH with the sample.

### Statistical analysis

To assess the effects of year, location, and their interactions, a combined analysis of variance was performed. Mean comparisons for traits at the three harvest dates were conducted using Duncan’s multiple range test at a 1% significance level using SPSS v.16. software. Based on these evaluations, the optimal harvest time for wonderful pomegranate in the studied regions was determined.

## Data Availability

All data supporting the findings of this study are available within the paper .
